# Effectiveness of low-dose doxycycline (LDD) on clinical symptoms of Sjögren's Syndrome: a randomized, double-blind, placebo controlled cross-over study

**DOI:** 10.1186/1477-5751-6-11

**Published:** 2007-12-31

**Authors:** Hubertus Seitsalo, Raija K Niemelä, Magdalena Marinescu-Gava, Tuija Vuotila, Leo Tjäderhane, Tuula Salo

**Affiliations:** 1 Institute of Dentistry, University of Oulu, PO BOX 5281, 90014 Oulu, Finland; 2 Oulu University Hospital (OUH), PO Box 50, 90029 OYS, Finland; 3 Division of Rheumatology, Department of Internal Medicine, Oulu University Hospital (OUH), PO Box 20, 90029 OYS, Finland; 4 Institute of Dentistry, University of Helsinki, and Department of Oral and Maxillofacial Diseases, Helsinki University Central Hospital (HUCH), PO BOX 14, 00014 University of Helsinki, Finland

## Abstract

**Background:**

Matrix metalloproteinases (MMPs) are proteolytic enzymes that may contribute to tissue destruction in Sjögren's syndrome (SS). Low-dose doxycycline (LDD) inhibits MMPs. We evaluated the efficacy of LDD for the subjective symptoms in primary SS patients.

This was a randomized, double blind, placebo controlled cross-over study. 22 patients were randomly assigned to receive either 20 mg LDD or matching placebo twice a day for 10 weeks. The first medication period was followed by 10-week washout period, after which the patient received either LDD or placebo, depending on the first drug received, followed by the second washout period. Stimulated saliva flow rates and pH were measured before and after one and ten weeks of each medication and after washout periods. VAS scale was used to assess the effect of LDD and placebo on following six subjective symptoms: xerostomia; xerophtalmia; difficulty of swallowing; myalgia; arthralgia; and fatigue. The effect was evaluated for each medication and washout period separately.

**Results:**

Overall, the effects of medications on subjective symptoms were minor. Wilcoxon test demonstrated increased fatigue with LDD during medication (p < 0.05). The differences may, however, reflect normal fluctuation of symptoms in SS patients.

**Conclusion:**

LDD may not be useful in reducing the primary SS symptoms.

## Background

Sjögren syndrome (SS) is a slowly progressing autoimmune rheumatic disease with unknown etiology [1]. It is associated with symptoms of xerostomia and xerophtalmia, and mononuclear cell infiltration of exocrine glands. Arthralgia, myalgia, Raynaud's phenomenon and fatigue are the most common systemic manifestations of SS. SS can be divided into primary and secondary forms. Primary SS is defined by the presence of salivary and lacrimal gland involvement as a sole systemic disorder. The course of the disease is often stable with slow initiation and progression of sicca symptoms. Primary SS is a separate disease entity, while secondary SS is concurrent with another rheumatic disease [1]. The prevalence of primary SS is estimated to be 0.6–4.0% of the world's population. About 90% of the patients are women, usually in their fifth or sixth decade. Because of the slow development of the symptoms, it is generally underdiagnosed [2-4].

Matrix metalloproteinases (MMPs) constitute a family of zinc-containing endoproteinases with 23 members. The principal function of MMPs is the proteolytic degradation of connective tissue matrix proteins, and in concert they can degrade practically all extracellular matrix proteins [5]. MMPs participate in the physiologic tissue regeneration, as in the wound healing, but they are also involved in the pathological conditions such as periodontitis, rheumatoid arthritis, other inflammatory diseases, and in the growth of tumors and cancer [5-9]. MMP expression and catalytic activity are increased in tissue samples from SS patients [10,11] and correlate with the severity of the disease and structural and functional glandular changes [10]. Primary SS patients exhibit increased plasma MMP-9 levels, which has been suggested to indicate definite primary SS [12]. SS patients' saliva contains increased concentrations of at least MMP-9, which is at least in part of glandular origin [13-15]. The increased salivary MMP-9 levels in relation to the endogenous tissue inhibitor of matrix metalloproteinase-1 (TIMP-1) have also been shown in primary SS patients [15].

The effect of medication targeting the potential factors behind the pathogenesis of SS on subjective symptoms has recently been a focus of interest in several studies [16,17], but the results have not been very promising. Due to their tissue-destructive capacity in chronic inflammatory diseases MMPs have been suggested as a potential target of action in the treatment of SS [13], including salivary glands [18,19].

Tetracyclines are antimicrobial agents, inhibiting also MMPs with a mechanism which is independent from their antimicrobial effect [20]. Doxycycline given in low doses (low-dose doxycycline, LDD) decreases significantly MMP activity by multiple mechanisms in the inflammatory diseases with no noticeable antimicrobial effects [21].

Based on the earlier studies a hypothesis was formed that through its MMP-inhibitory action, LDD could be effective in treatment of SS by decreasing the tissue damage and therefore also the subjective symptoms of the patients. The aim of this study was to evaluate the effectiveness of LDD on clinical symptoms of SS in a randomized, double-blinded, placebo-controlled clinical cross-over trial.

## Results

**Figure 1:**
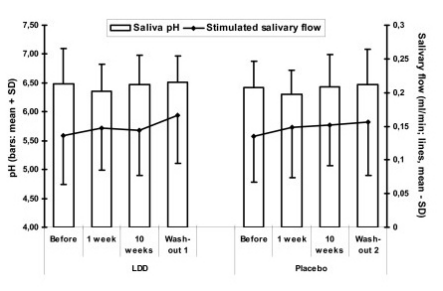
**Stimulated saliva pH and salivary flow rate during the experiment**. There were no statistically significant differences between any of the time points either in pH or salivary flow values (Wilcoxon sign-rank test).

Seventeen subjects out of 22 patients included at the onset of the study (77%) completed the study and provided the VAS score data after each medication and washout period. The stimulated salivary flow rates and pH were low compared to normal reference values (salivary flow > 0,7 ml/min, pH 7,3 for stimulated saliva) (Figure 1). No statistically significant differences were observed between any time points (Wilcoxon signed ranks test).

**Figure 2:**
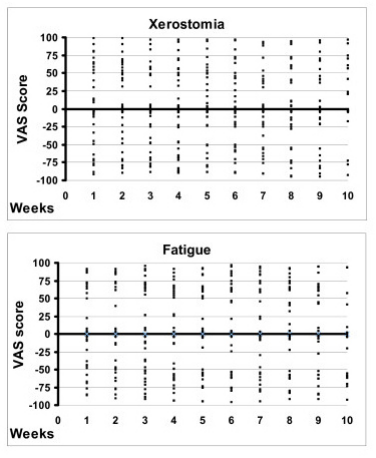
**Plot figures of individual VAS scores**. Plot figures of individual VAS scores of xerostomia and fatigue during the LDD medication period (expressed as positive values above x-axis) and respective placebo period (expressed as negative values below the x-axis). No apparent effect on any of the subjective symptoms recorded was seen during either one of the medications. Similar distribution of scores was observed for the other symptoms (data not shown).

In Figure 2 the VAS score distribution of the LDD and placebo medication periods for two symptoms (xerostomia and fatigue) are presented as descriptive plot figures. The plots demonstrate remarkably similar distribution of VAS scores for LDD and placebo throughout both medication periods, with no apparent changes during either medication or between the medications. With the other symptoms evaluated, similar distribution of scores was observed (data not shown).

**Figure 3:**
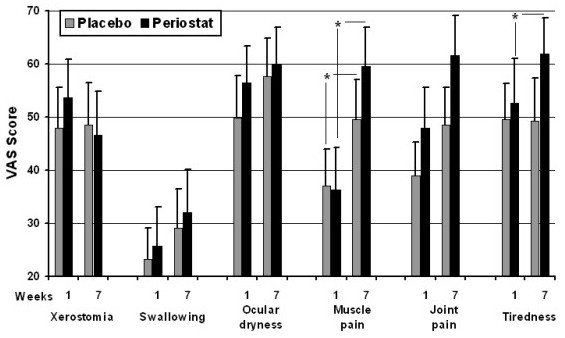
**VAS scores of measured subjective symptoms in weeks one and seven**. * indicates statistically significant differences between the observation time points (p < 0.05; Wilcoxon signed test).

Comparison of the VAS scores between the pre-medication and seven-week medication demonstrated statistically significantly higher values for myalgia after seven weeks of both LDD and placebo (Figure 3). Comparison of the VAS scores between LDD and placebo after seven weeks of medication demonstrated statistically significantly higher VAS scores for fatigue after LDD medication (Figure 3) (p < 0.05 in both cases: Wilcoxon signed ranks test).

### Post-experimental interview

For the post-experimental telephone interview, 16 patients were reached. Five patients had experienced significant alleviation of symptoms during one of the medication periods. Three patients had felt the benefits during the LDD medication and two had felt the benefits during placebo medication.

## Discussion

In spite of tissue destructive nature of primary SS, the subjective symptoms are the most significant handicap. Therefore development of palliative treatment strategies has long been an object of interest. Local treatments, such as artificial saliva or lozenges, are insufficient and provide only temporary relief [22,23]. Pilocarpine is the only widely accepted systemic treatment method that has been shown to be effective [24], but the severe side effects and unpredictability of clinical response limit its use [25]. Cevimeline, a quinuclidine derivative of acetylcholine receptors, has been indicated to alleviate subjective symptoms without significant side effects [22], but further research is needed before the wide use of this drug can be substantiated. Therefore a need for effective, safe and practical systemic treatment measures to alleviate the subjective symptoms associated with primary SS still exists. Ideally, relief of subjective symptoms should occur concomitantly with reduced tissue destruction. As low-dose doxycycline has recently been shown to reduce both the recurrence of oral aphthous ulceration and subjective experience of pain in recurrent oral aphthous ulceration (ROAU) patients [26], the palliative effect of LDD also in SS patients could be expected.

The low salivary flow rate and low saliva pH were expected, as values of this kind are normally found in SS patients with established disease. Measuring the unstimulated salivary flow rate proved impossible due to practically zero unstimulated saliva secretion in most patients. The findings indicate the severe condition of SS in the subjects involved in the study.

Overall, the changes in the subjective symptoms within the LDD or placebo medication or washout periods were small and most likely reflect the usual fluctuation of symptoms rather than true differences caused by medication. Considering the subjective symptoms that exhibited statistically significant differences within specific medication or washout, only the increase in fatigue with LDD medication seems possibly clinically significant (Figure 3). Muscle pain as a possible side effect has been described in the Periostat Prescribing Information sheet, but the percentage of incidence (1%) is lower than with the placebo group (3%). Also the other pain-related adverse reactions with Periostat medication are low. Considering the fact that statistically significant rise in myalgia VAS scores was detected also in the LDD washout period and in placebo medication period when comparing the first and seventh week VAS scores (Figure 3), it is possible that even with the sharp increase in VAS pain score after 5^th ^week of Periostat medication the finding may be a coincidence. This is supported by the post-experimental interview, which clearly indicated that there was no overall effect on subjective symptoms by LDD. On the other hand, taking into consideration the multi-dimensional subjective symptomology of SS patients, with mostly unknown pathology behind the symptoms, it is possible that MMP inhibition might in some way increase the subjective feeling of myalgia. The lack of clinically significant findings in this study may also be affected by the low numer of patients (22 patients) involved, even though all the patients in the patient records of Northern Ostrobothnia Hospital District (Oulu University Hospital, covering 12 % of Finland's surface area) with SS diagnosis were included into screening. Another study with larger patient population, requiring multi-center study, would be needed for the final conclusion in this matter. However, since the positive effects of LDD medication on SS subjective symptoms seem to be minor, larger-scale studies with SS patients, at least the patients with established disease as in this study, do not seem justified.

## Conclusion

MMP inhibition with LDD was not effective in alleviating the subjective symptoms of primary SS patients with already established disease. The finding is concurrent with the other studies aiming at the reduction of symptoms by directing the systemic medication against potential pathogenetic factors [16,17], with the possible exception of cemiveline hydrochloride [22]. This study does not exclude the possibility of beneficial effect of MMP inhibition on slowing down or inhibiting the MMP-mediated tissue destruction at least in secretory glands [10,11,13,18,19]. However, since the disease is usually diagnosed only after advanced gland tissue destruction has already occurred, sensible and specific diagnostic methods to screen the primary SS patients before the development of subjective symptoms would be needed before the preventive treatment can be provided. Also, recent study indicating that MMP-9 may actually have a protective role against eruption of purpura and development of autoantibody reaction in primary SS [12], signify the importance of fundamental understanding of MMPs in SS pathogenesis before interceptive treatment can be justified in symptomless patients.

## Methods

Detailed description of the patient inclusion and exclusion criteria has been presented previously [27]. Briefly, consecutive outpatients with existing diagnosis of primary SS from the Department of Rheumatology in Oulu University Hospital, Oulu, Finland, constituted the patient group. For the diagnosis of primary SS the patient had to fulfil the revised European Community proposed criteria [28]. Approval for the study protocol was obtained from the Oulu University Hospital Ethical Committee and from the National Agency for Medicines. All the patients gave their written informed consent. A total of 44 patients were screened, 27 patients were qualified to participate and 22 patients chose to enter the study. All the patients were women.

This was a randomized, double blind, placebo-controlled clinical trial, and the patients were randomized at the baseline. Randomization was performed by use of a computer-generated list, and the drug and placebo were indistinguishable in appearance, smell and taste. Neither the person in charge of clinical protocol of the study nor the patients were aware of the treatment assignments.

**Figure 4:**
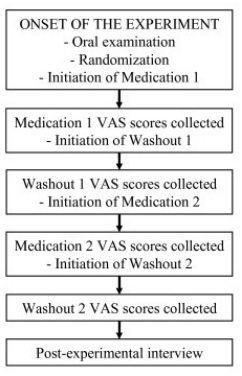
**The outline of the study protocol and time-table**. After each medication period or washout period the VAS score sheets were collected and new sheets for the subsequent period were provided for the patient. Post-experimental interview was performed to evaluate whether the patients had felt overall subjective alleviation of symptoms during the medication.

At the onset of the study, the patients underwent a thorough oral and dental examination as a part of normal dental treatment. One of the patients had gingivitis. Some patients had a few secondary caries lesions or primary cervical caries lesions. The normal dental treatment was provided at the onset of the study according to the need. The patients were randomly assigned to receive either low-dose doxycycline (20 mg doxycycline; Periostat^®^; further referred as LDD) or matching placebo (both from CollaGenex Pharmaceuticals, Inc. Newtown, PA, USA) twice a day for a period of 10 weeks (Medication 1; see Figure 4). The first medication period was followed by 10-week washout period (Washout 1; Figure 4), after which the patient received either LDD or placebo, depending on the first drug received (Medication 2; Figure 4), followed by the second washout period. Stimulated and unstimulated saliva was collected, and flow rate and pH were measured at the onset and after one and 10 weeks of each medication period, as well as after 10-week washout periods.

To assess the effect of LDD and placebo on subjective symptoms, six 100-mm Visual Analog Scale forms (VAS; form identical to those used to describe pain) were used to describe the intensity of the following symptoms: xerostomia; xerophtalmia; difficulty of swallowing; myalgia; arthralgia; and fatigue. The patients were instructed to provide a VAS rating every week at the same time of the week for each of the six symptoms, using a range from 0 for the total absence of symptom to 100 for worst imaginable symptom. After each medication and washout period the VAS score sheets were collected (Figure 4) and new score sheets provided for the next period.

Six months after the experiment the patients were contacted by telephone and asked for their subjective opinion whether the drug provided any help in general for their subjective symptoms or not (post-experimental interview) (Figure 4).

### Statistical analysis

SPSS for Windows Release 11.5.1 (SPSS Inc., Chicago, IL, USA) was used for the statistical analysis. Since the number of the marked scores decreased towards the end of each medication and washout period (because of apparent reduction of co-operation towards the end of each period), the statistical analysis was only extended to the 7th week of each period. Wilcoxon signed ranks test for two related variables was used to analyse the differences between the pre-medication and 7-week medication samples within both medication regimens, and between the values after 7-week LDD and 7-week placebo medications, to evaluate the possible effect of LDD on subjective symptoms. The same test was also used to examine the differences between the pre-medication levels, to exclude the effect of possible differences at the starting point of the medication regimens.

## List of abbreviations

LDD: Low-dose doxicycline;

SS: Sjögren's Syndrome;

MMP: Matrix metalloproteinases.

## Competing interests

The author(s) declare that they have no competing interests.

## Authors' contributions

HS was responsible for the handling of the subjects and collection of the samples, participated into data analysis and writing of the manuscript. RKN participated in the design of the study, collection of the patient material and drafting of the manuscript. MM-G participated in the handling of the collection and analysis of the samples. TV participated in the design of the study. LT participated in preliminary data analysis, performed the statistical analysis and drafting of the manuscript. TS conceived of the study, participated in its design, coordination, funding, data-analysis and drafting of the manuscript. All authors read and approved the final manuscript.
